# Operation for Club Foot

**Published:** 1842-05

**Authors:** 


### OPERATION FOR CLUB FOOT.

The communication from Samuel Silsbee, M. D., of Cincinnati, of which the following is an extract, would have been noticed some time since, but that it was mislaid. The Doctor was himself the subject of the operation—his age about 24 years.

"On the 22nd of February, 1840, the tendo Achilles was divided with but little pain, and the loss of a single drop of blood; and the foot was placed immediately in a state of extension. On the 25th it was found necessary to divide the plantar fascia, which was done, but unlike the former operation, was attended with the most acute pain. On the 27th, scarcely more than a scar was visible of the operation. I was confined to my couch, and my whole time and that of many interested friends, was occupied in manipulating with the foot by frictions, extensions, emollients, &c. The progress we made was almost incredible, and such was the reproductive powers of nature, under a careful system of dietetics, conjoined with youth and good health, that about the 28th of March, the divided extremities of the tendon had reunited by new substance to the distance of 1½ inches, and in such a perfect manner that the point of division could not be distinguished. But in the progress of extension we were obliged to break the newly formed tendon. In seven weeks from the time of operation, the plantar surface of the foot could nearly be placed upon the floor; and in ten weeks the foot was placed in a straight leather boot, with stiff back, and straps to keep it in a state of extension, which apparatus, with some modifications, I have retained ever since.

When in this boot, I commenced using my foot; but for the first two months, I suffered most infinite torture from the pressure upon the phalangeal extremities of the metatarsal bones. This I presume time will obviate; in fact it has been much palliated by making holes in the sole of the boot, and by the free use of padding.

I also found, in spite of all the ingeniously contrived inventions upon which I spent much time, and no little money, including many of those most approved by eminent surgeons, exhausting my own patience and that of my workmen, that so much force was continually required to keep the foot in position, and such unequal pressure was unavoidably made on various points, as to keep me in a constant state of annoyance, amounting at times to actual suffering. From the same cause the development of the foot (if it were so inclined) was prevented. But the most unpleasant result of my experiment which I had to contemplate, was, that as my foot increased in strength (which it did, so much so that I limped along slowly, but without pain), my ankle grew stiff and immovable, in spite of warm baths, frictions, constant use, &c. This then was my condition 9 months after the operation, previous to which I was able to compete with any of my age in pedestrianism, and as far as my *foot* was concerned, equalled in agility most of my fellows. Now to whom, or to what can this failure be attributed? I cannot, nor will any one, ascribe it to inattention, ignorance, or mismanagement on my part, for surely none could feel more personally interested in a favorable result than myself, and few operations commenced under more auspicious circumstances than did mine. That it is a *failure,* however, no one who sees my diminutive, misshapen libel upon a legitimate foot, and witnesses my decrepit progression, can for a moment doubt." D.

